# Supplementary material for: Digital PCR characterizes epithelial cell populations in murine duodenal organoids
Source: PLoS One. 2025 Mar 13;20(3):e0319701. doi: 10.1371/journal.pone.0319701 (PMC11906084; doi:10.1371/journal.pone.0319701)
Supplement: S1 Supporting information — (DOCX) [file pone.0319701.s001.docx]

**S1 Supporting information**

***Chelating buffer***

Freshly prepare 100 ml of chelating buffer (5X):

Na_2_HPO_4_ (28 nM), KH_2_PO_4_ (40 nM), NaCl (480 nM), KCl (8 nM), Sucrose (220 nM) and D-Sorbitol (274 nM).

Working solution (1X) is prepared in Milli-Q water added with 0.5M 1,4-dithiothreitol (DTT) and 0.5M ethylenediamine tetra acetic acid (EDTA), pH 8.0.
