# Supplementary material for: Digital PCR characterizes epithelial cell populations in murine duodenal organoids
Source: PLoS One. 2025 Mar 13;20(3):e0319701. doi: 10.1371/journal.pone.0319701 (PMC11906084; doi:10.1371/journal.pone.0319701)
Supplement: S1 Table — (DOCX) [file pone.0319701.s002.docx]

**S1 Table. Quantification of target sequences of duodenal cell populations**

| Cell population | Marker | Copies /µl | | | | | | | | |
| --- | --- | --- | --- | --- | --- | --- | --- | --- | --- | --- |
|  |  | **Early passage** | | | **Intermediate passage** | | | **Late passage** | | |
|  |  | **M1** | **M2** | **M3** | **M1** | **M2** | **M3** | **M1** | **M2** | **M3** |
| Stem cells | LGR5 | 1.518 | 32.7 | 555.3 | 0.385 | 67.9 | 1.396 | 6.256 | 95.45 | 40.08 |
| Stem cells | SOX9 | 11.26 | 1201.3 | 739.6 | 54.75 | 2081.4 | 52.11 | 97.54 | 487.3 | 69.33 |
| Proliferating cells | PCNA | 3350.9 | 1513.6 | 5656.5 | 409.1 | 999.8 | 888.4 | 2610.2 | 2996.1 | 423.4 |
| Goblet cells | MUC2 | 261.9 | 2566.3 | 2.128 | 2.129 | 95.15 | 268.8 | 2.28 | 44.84 | 12.1 |
| Tuft cells | DCLK1 | 5.562 | 28.73 | 70.16 | 29.24 | 32.87 | 1.42 | 2.237 | 90.78 | 7.241 |
| Enteroendocrine cells | ChA | 7.106 | 13.31 | 457.5 | 1.391 | 12.77 | 632 | 194.3 | 46.54 | 140.3 |
| Paneth cells | LYS | 175 | 51.63 | 48.08 | 40.48 | 59.4 | 43.25 | 4.18 | 117.3 | 2.243 |
| Epithelial cells | EPCAM | 8979.2 | 3612.4 | 10446.3 | 1036.3 | 1628 | 1635.4 | 1163.9 | 4155.1 | 551.2 |
| Microvilli | VILLIN | 377.9 | 755.9 | 533.6 | 898.7 | 868.8 | 75.68 | 150 | 84.67 | 22.63 |
| Endogenous control | GAPDH | 3348.5 | 12467.9 | 19209.6 | 3966.9 | 7108.2 | 5675.7 | 8206.7 | 23309.6 | 3527.9 |
