# Supplementary material for: Digital PCR characterizes epithelial cell populations in murine duodenal organoids
Source: PLoS One. 2025 Mar 13;20(3):e0319701. doi: 10.1371/journal.pone.0319701 (PMC11906084; doi:10.1371/journal.pone.0319701)
Supplement: S2 Table — (DOCX) [file pone.0319701.s003.docx]

**S2 Table. Primer sequences and NCBI gene accession numbers**

| **Cell type** | **Gene**  **abbreviation** | **Genbank**  **Accession number** | **Gene name/ Primers sequence** | | **Product size**  **(bp)** |
| --- | --- | --- | --- | --- | --- |
| Stem cells | LGR5 | 14160 | **Leucine-rich repeat containing G protein-coupled receptor 5** | | |
|  |  |  | **5’** | CAGTGTTGTGCATTTGGGGG | 178 |
|  |  |  | **3’** | GAAGGGCCTTCAGGTCTTCC |  |
|  | SOX9 | 20682 | **SRY (sex determining region Y)-box 9** | | |
|  |  |  | **5’** | TCCCCGCAACAGATCTCCTA | 157 |
|  |  |  | **3’** | AGGTGGAGTAGAGCCCTGAG |  |
| Proliferative | PCNA | 18538 | **Proliferating Cell Nuclear Antigen** | | |
|  |  |  | **5’** | TGCACGTATATGCCGAGACC | 96 |
|  |  |  | **3’** | CCAAGCTCTCCACTTGCAGA |  |
| Tuft | Dclk1 | 13175 | **Doublecortin-like kinase 1** | | |
|  |  |  | **5’** | CCCCTGGTAGTCAGCTCTCT | 93 |
|  |  |  | **3’** | GAGAGATCCTCTGCTTCCGC |  |
| Goblet | Muc2 | 17831 | **Mucin 2** | | |
|  |  |  | **5’** | CCTGAAGACTGTCGTGCTGT | 100 |
|  |  |  | **3’** | GGGTAGGGTCACCTCCATCT |  |
| Mature epithelial | EPCAM | 17075 | **Epithelial Cell Adhesion Molecule** | | |
|  |  |  | **5’** | CCTGAGAGTGAACGGAGAGC | 132 |
|  |  |  | **3’** | GACACCACCACAATGACAGC |  |
| Paneth cells | Lyz1 | 17110 | **Lysozyme** | | |
|  |  |  | **5’** | TGACTCTGGGACTCCTCCTG | 113 |
|  |  |  | **3’** | CTTGACACCACGGTAGCCAT |  |
| Microvilli | Vil1 | 22349 | **Villin 1** | | |
|  |  |  | **5’** | TCCTGGCTATCCACAAGACC | 169 |
|  |  |  | **3’** | CTCTCGTTGCCTTGAACCTC |  |
| Enteroendocrine | Chga | 12652 | **Chromogranin A** | | |
|  |  |  | **5’** | GAAGTGCGTCCTGGAAGTCA | 98 |
|  |  |  | **3’** | TCCTCTCGTCTCCTTGGAGG |  |
| Housekeeping | GAPDH | 14433 | **Glyceraldehyde-3-phosphate dehydrogenase** | | |
|  |  |  | **5’** | GGCAAATTCAACGGCACAGT | 164 |
|  |  |  | **3’** | GCCTTCTCCATGGTGGTGAA |  |

**Antibodies**

| **Antigen** | **Host** | **Supplier** | **Catalog number** |
| --- | --- | --- | --- |
| ***Primary antibodies* (Dilution 1:100)** | | | |
| Muc-2 | anti-mouse | Abcam | ab11197 |
| Lysozyme | anti-mouse | Abcam | ab36362 |
| Villin-1 | anti-rabbit | Abcam | ab130751 |
| E-cadherin | anti-mouse | Abcam | ab231303 |
| Sox 9 | anti-rabbit | Abcam | ab182579 |
| LGR5 | anti-mouse | Abcam | ab273092 |
| Occludin | anti-rabbit | Invitrogen | PA5-30230 |
| ***Secondary antibodies* (Dilution 1:400)** | | | |
| Alexa flour 488 donkey | anti-mouse | Thermo Fisher | A21202 |
| Alexa flour 488 donkey | anti-rabbit | Thermo Fisher | A21206 |
| Alexa fluor 647 donkey | anti-mouse | Thermo Fisher | A31571 |
| Alexa fluor 647 donkey | anti-rabbit | Thermo Fisher | A31573 |
