# Supplementary material for: Digital PCR characterizes epithelial cell populations in murine duodenal organoids
Source: PLoS One. 2025 Mar 13;20(3):e0319701. doi: 10.1371/journal.pone.0319701 (PMC11906084; doi:10.1371/journal.pone.0319701)
Supplement: S3 Table — (DOCX) [file pone.0319701.s004.docx]

**S3 Table. Antibodies used for confocal microscopy detection of specific antigens**

| **Antigen** | **Host** | **Supplier** | **Catalog number** |
| --- | --- | --- | --- |
| ***Primary antibodies* (Dilution 1:100)** | | | |
| Muc-2 | anti-mouse | Abcam | ab11197 |
| Lysozyme | anti-mouse | Abcam | ab36362 |
| Villin-1 | anti-rabbit | Abcam | ab130751 |
| E-cadherin | anti-mouse | Abcam | ab231303 |
| Sox 9 | anti-rabbit | Abcam | ab182579 |
| LGR5 | anti-mouse | Abcam | ab273092 |
| Occludin | anti-rabbit | Invitrogen | PA5-30230 |
| ***Secondary antibodies* (Dilution 1:400)** | | | |
| Alexa flour 488 donkey | anti-mouse | Thermo Fisher | A21202 |
| Alexa flour 488 donkey | anti-rabbit | Thermo Fisher | A21206 |
| Alexa fluor 647 donkey | anti-mouse | Thermo Fisher | A31571 |
| Alexa fluor 647 donkey | anti-rabbit | Thermo Fisher | A31573 |
