# Supplementary figures and images for: Digital PCR characterizes epithelial cell populations in murine duodenal organoids
Source: PLoS One. 2025 Mar 13;20(3):e0319701. doi: 10.1371/journal.pone.0319701 (PMC11906084; doi:10.1371/journal.pone.0319701)

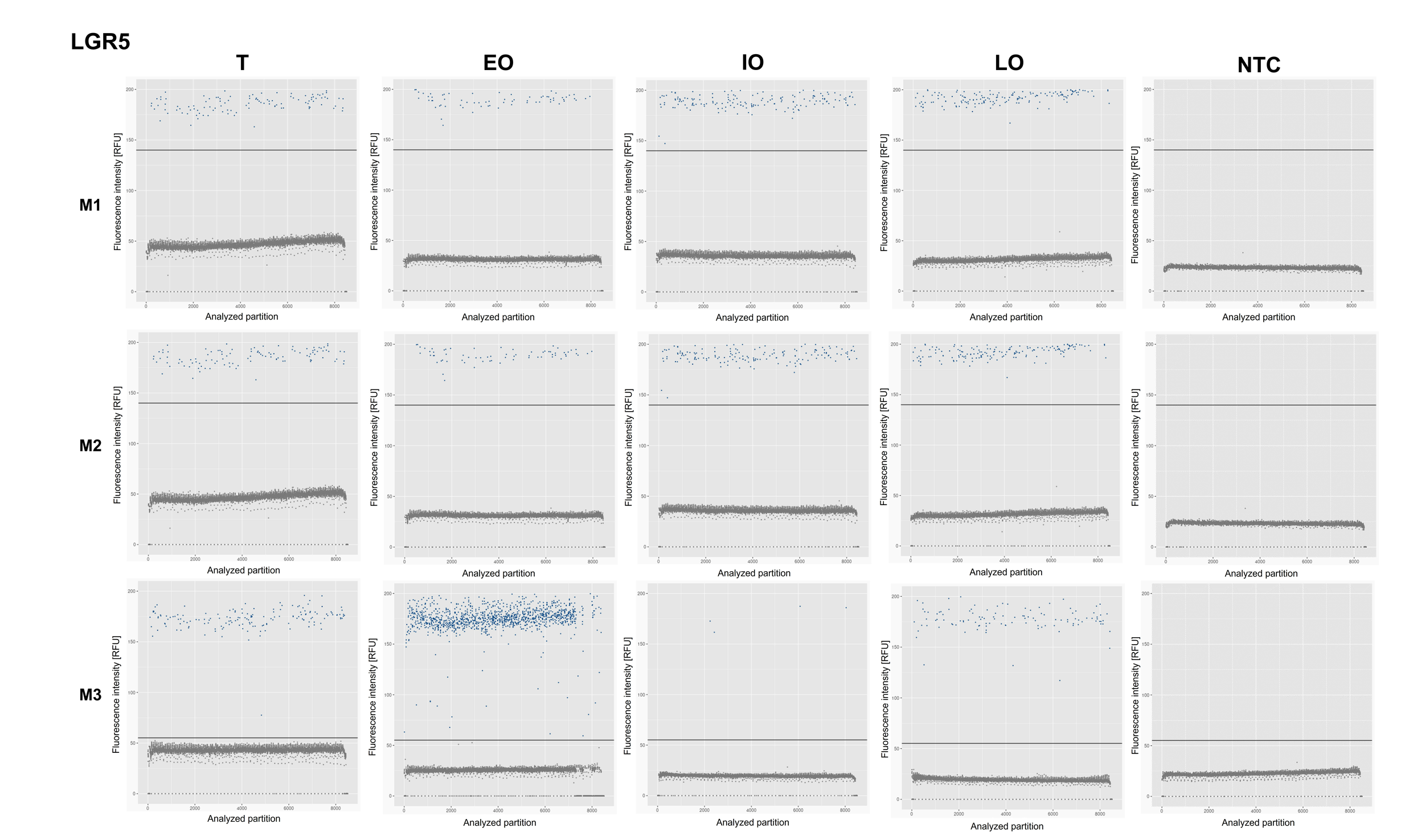

Supplement: S1 Fig — (TIF) [file pone.0319701.s005.tif]

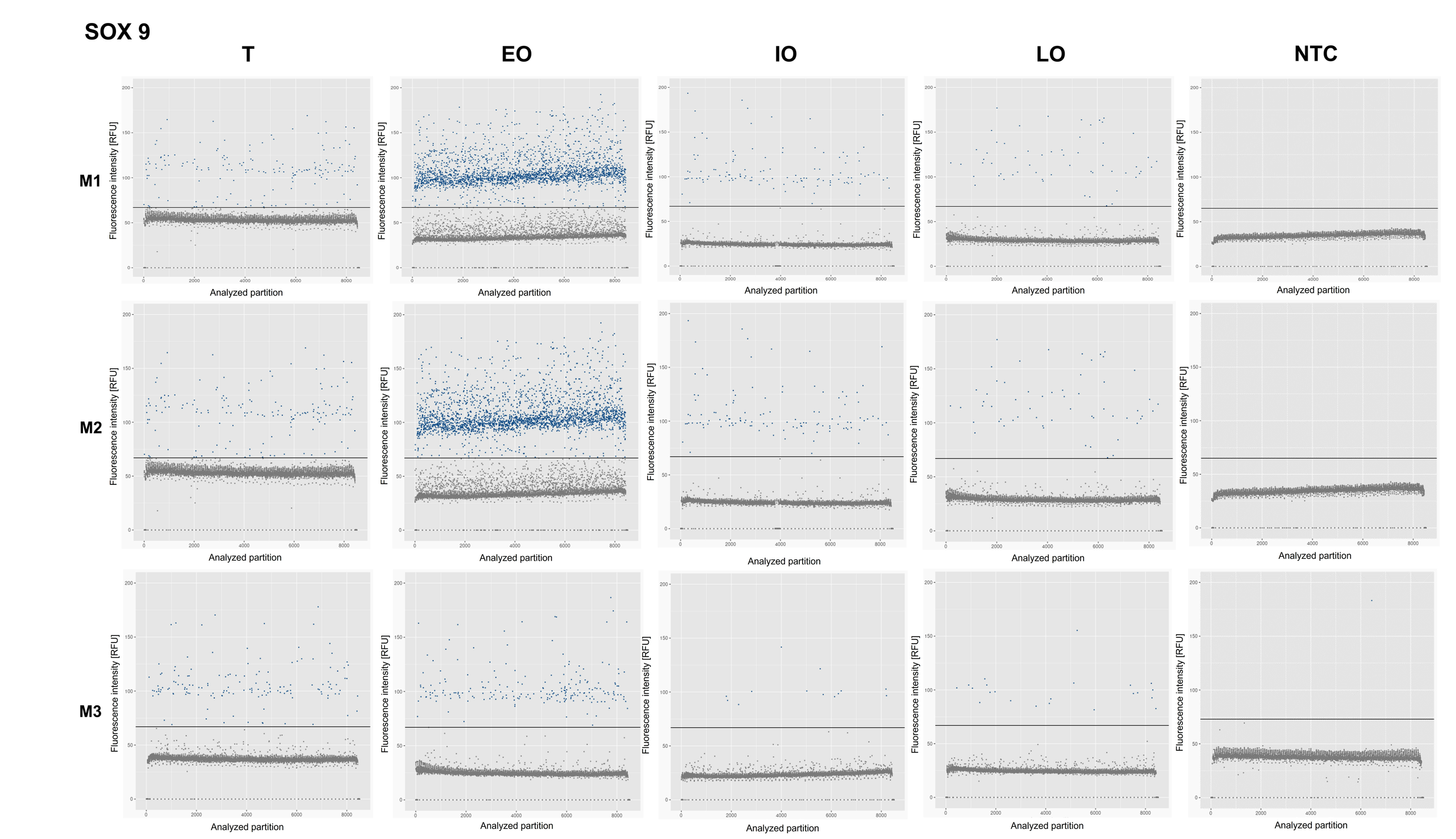

Supplement: S2 Fig — (TIF) [file pone.0319701.s006.tif]

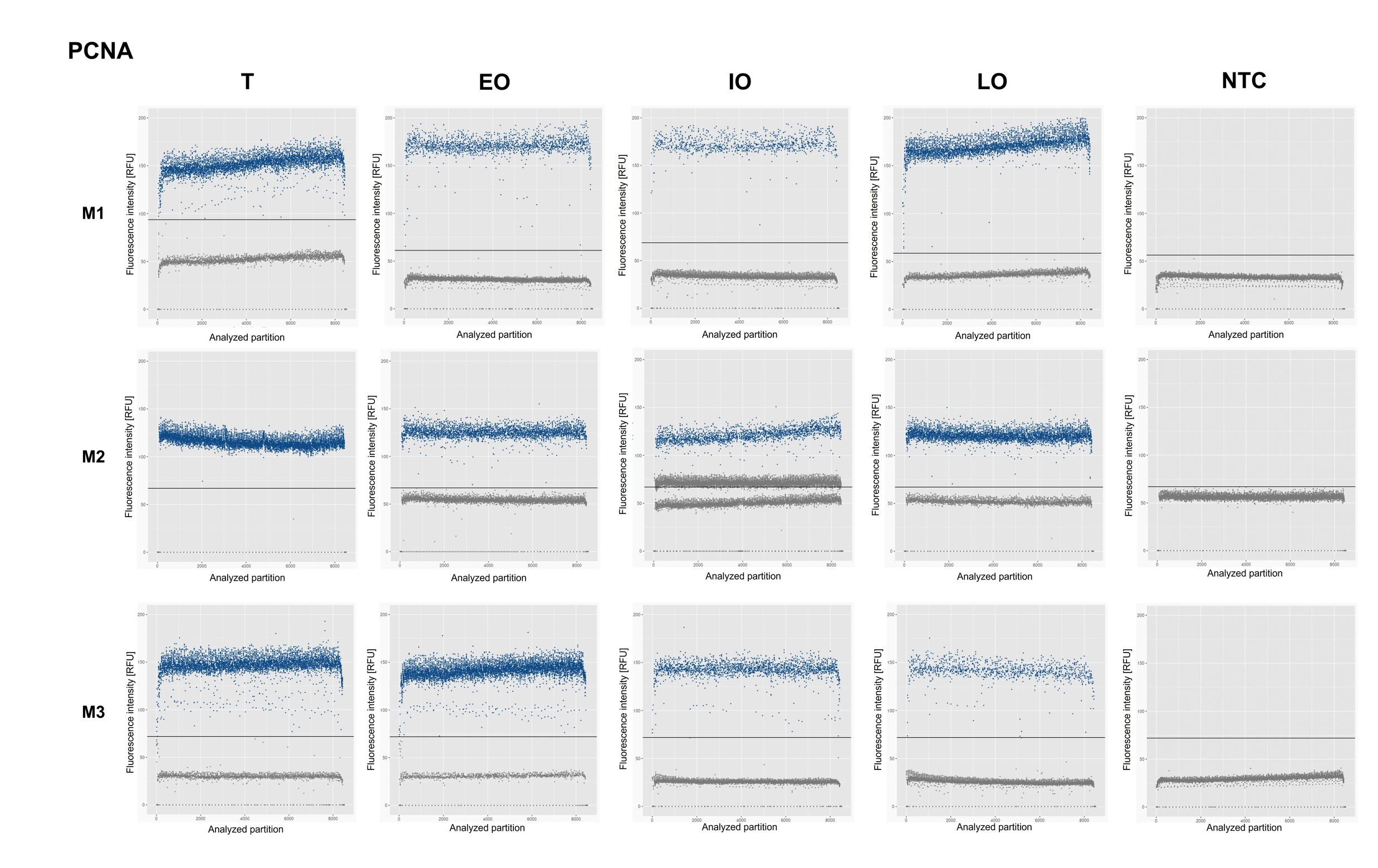

Supplement: S3 Fig — (TIF) [file pone.0319701.s007.tif]

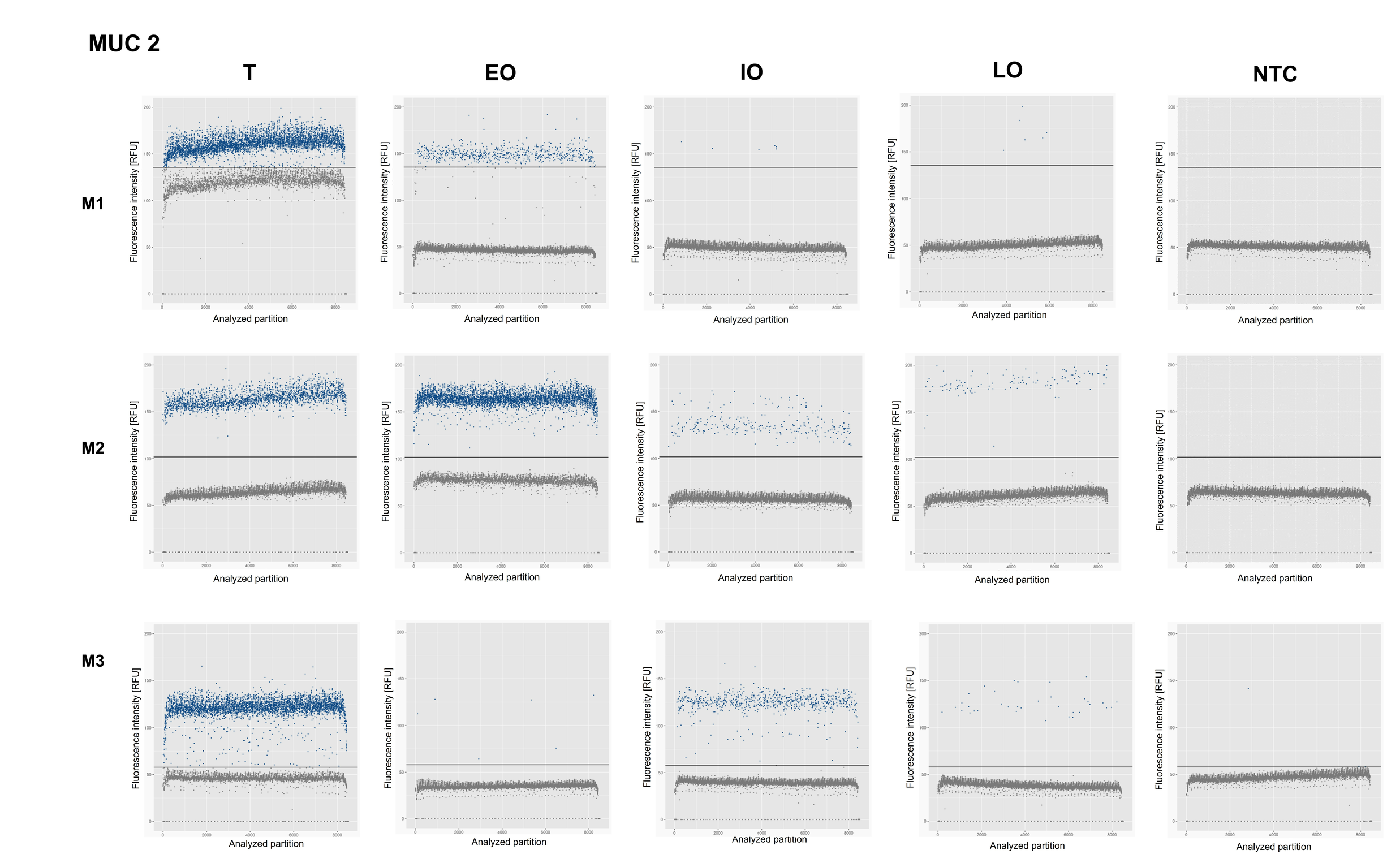

Supplement: S4 Fig — (TIF) [file pone.0319701.s008.tif]

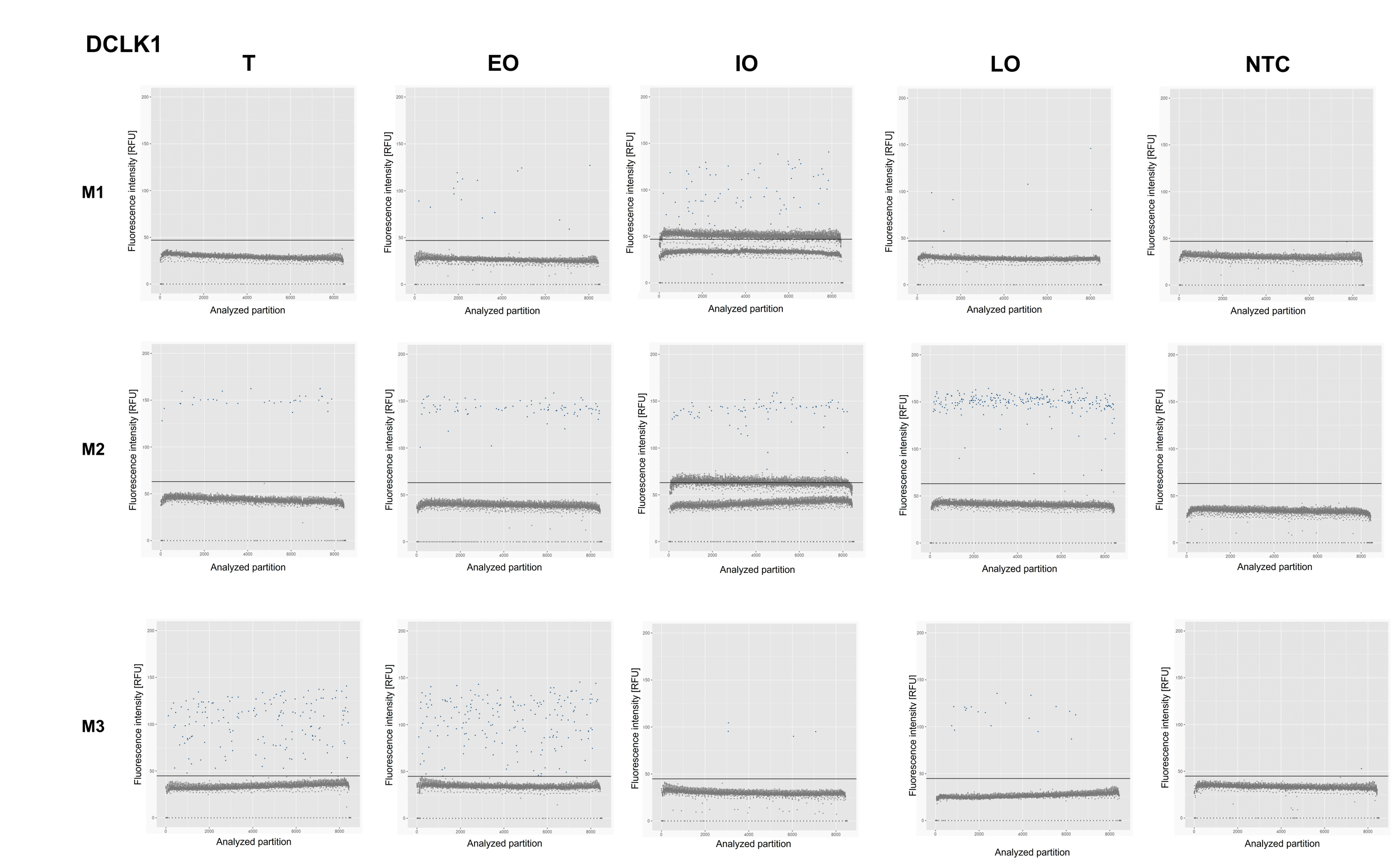

Supplement: S5 Fig — (TIF) [file pone.0319701.s009.tif]

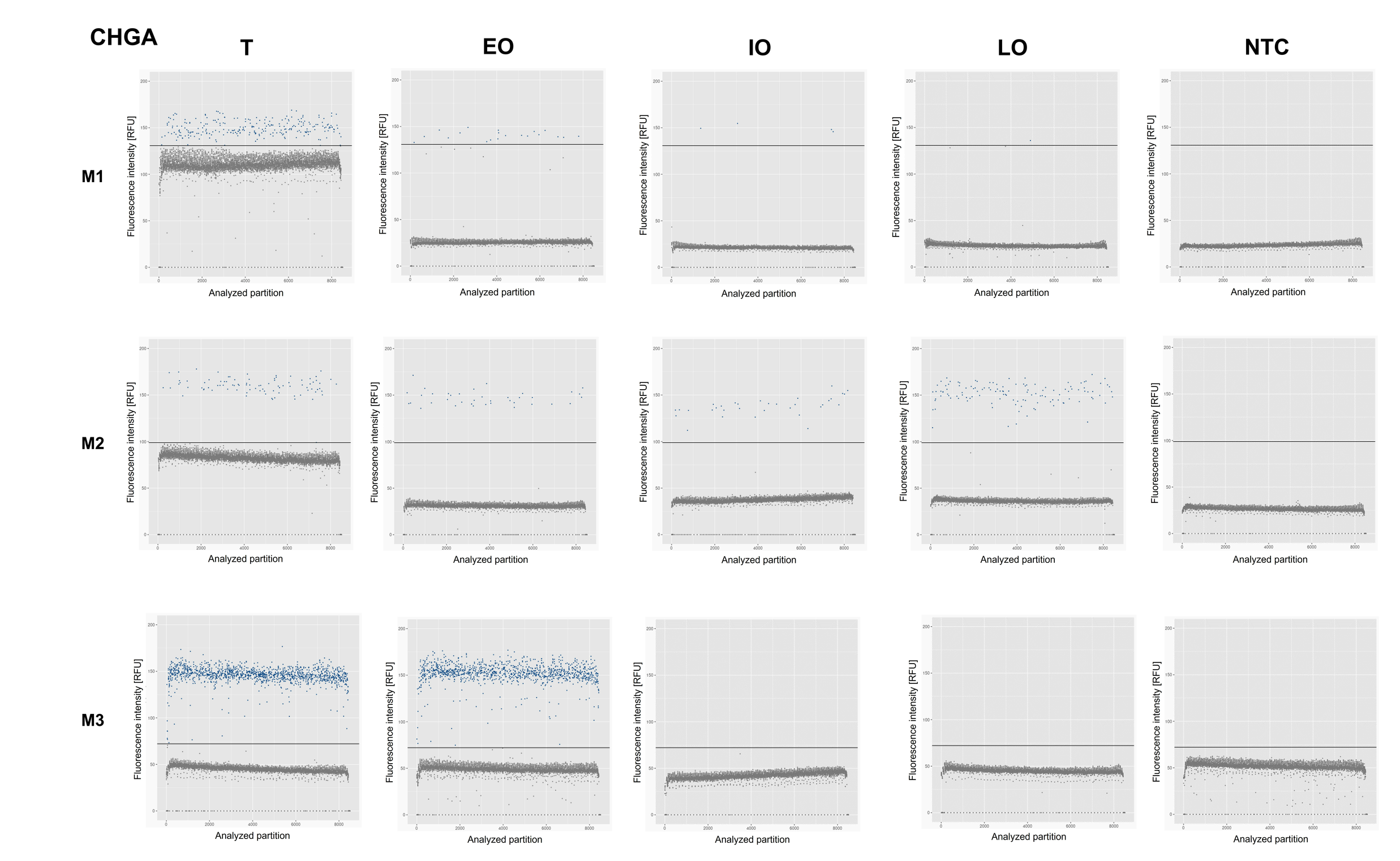

Supplement: S6 Fig — (TIF) [file pone.0319701.s010.tif]

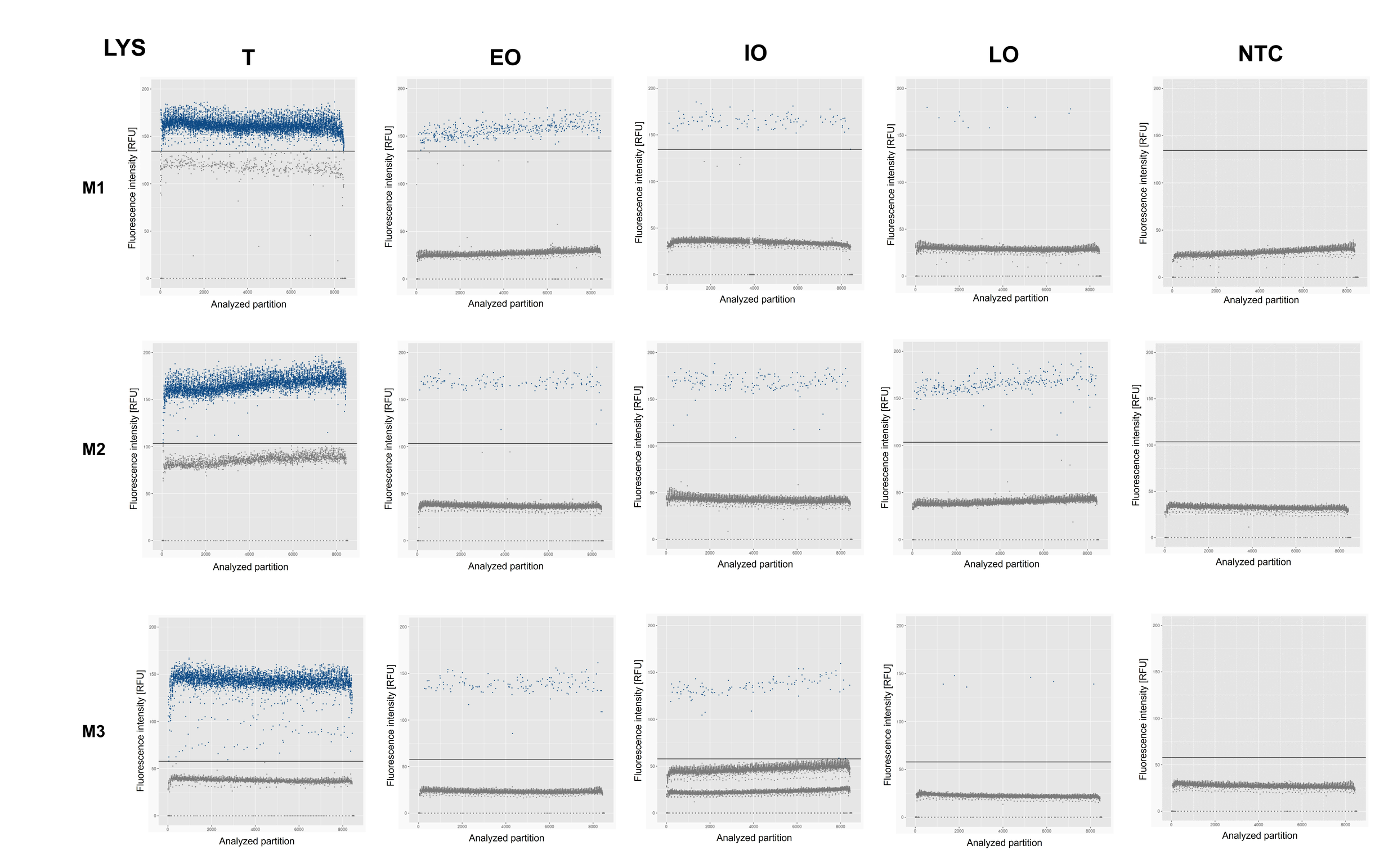

Supplement: S7 Fig — (TIF) [file pone.0319701.s011.tif]

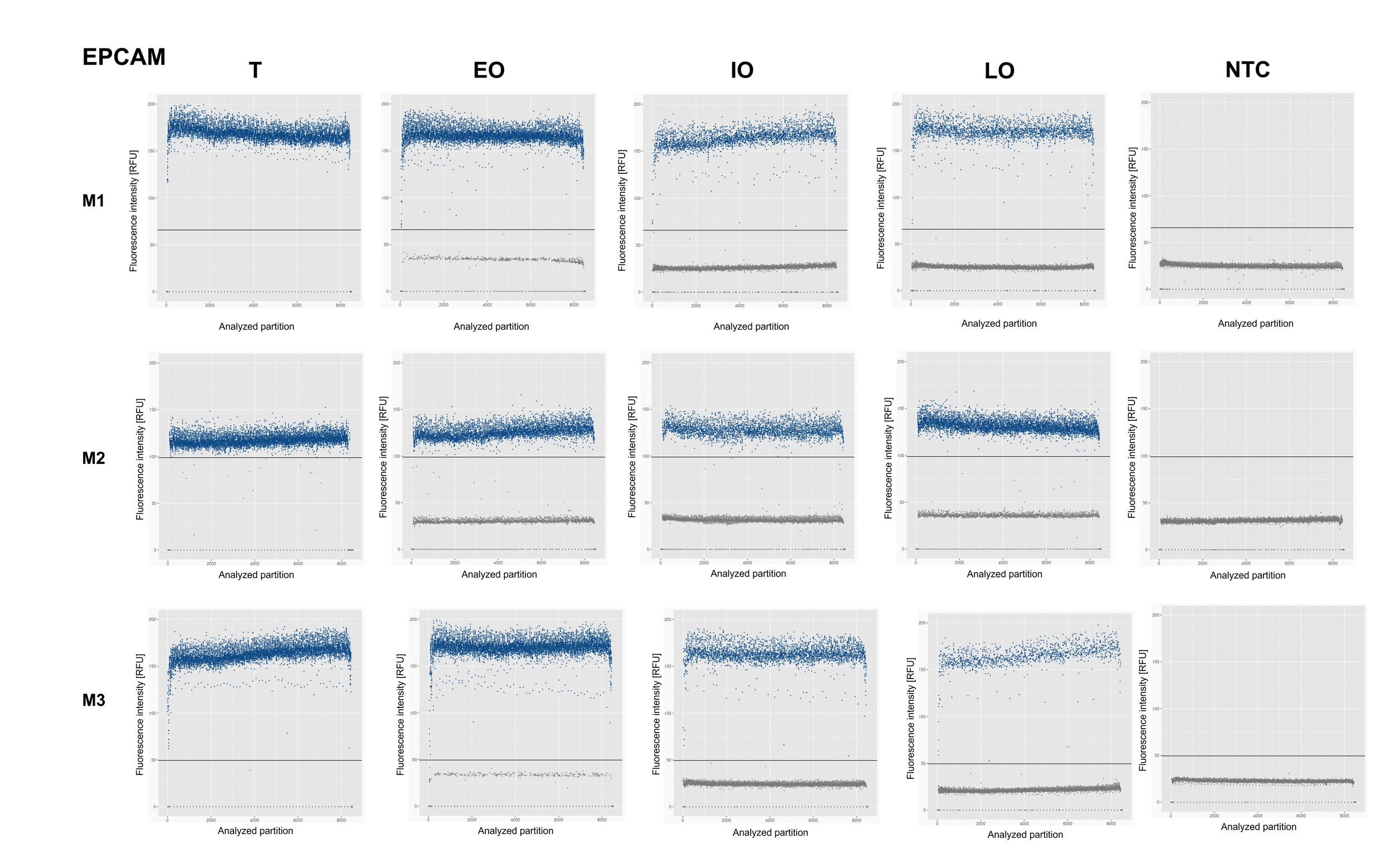

Supplement: S8 Fig — (TIF) [file pone.0319701.s012.tif]

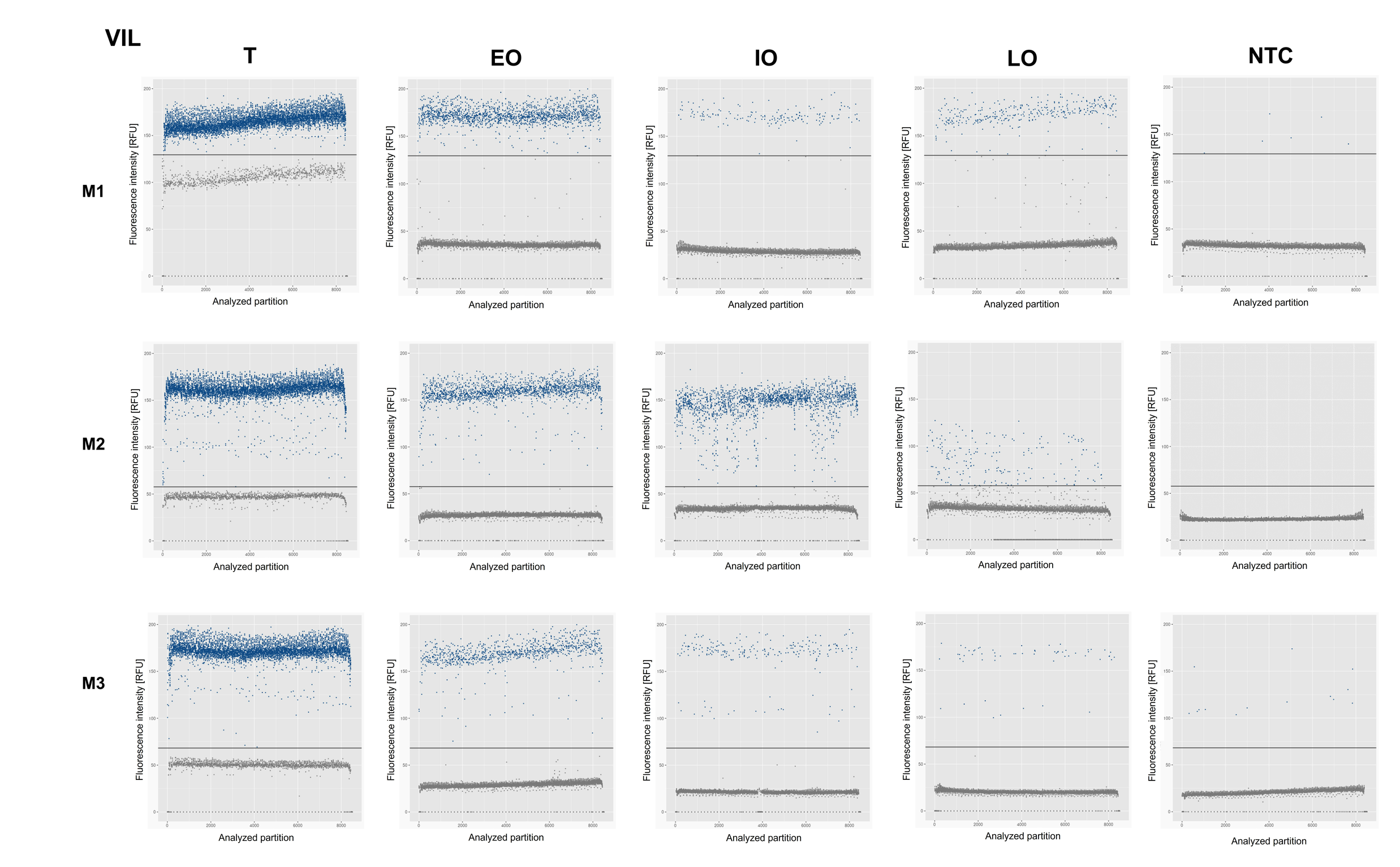

Supplement: S9 Fig — (TIF) [file pone.0319701.s013.tif]

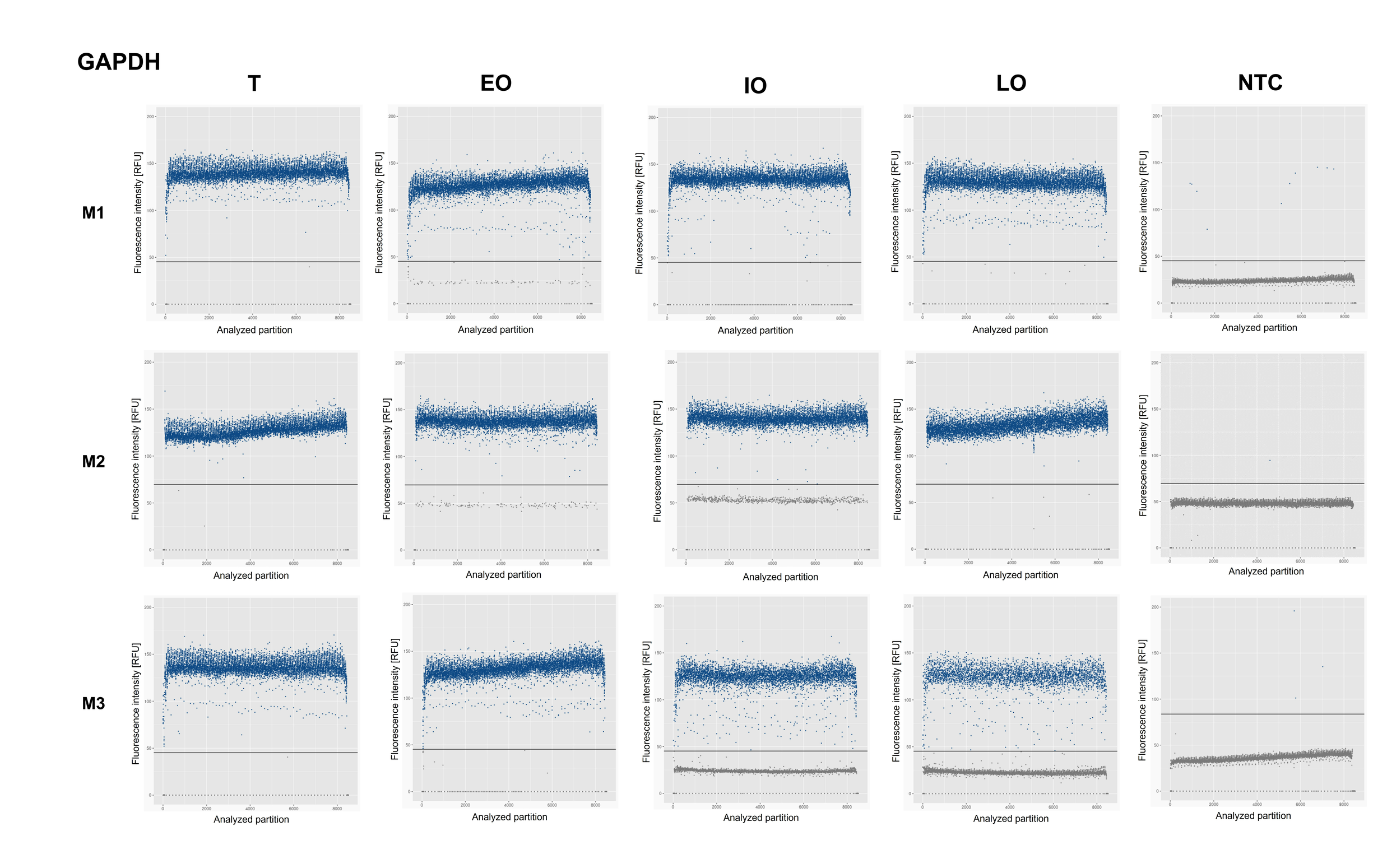

Supplement: S10 Fig — (TIF) [file pone.0319701.s014.tif]
