# Supplementary material for: Digital PCR characterizes epithelial cell populations in murine duodenal organoids
Source: PLoS One. 2025 Mar 13;20(3):e0319701. doi: 10.1371/journal.pone.0319701 (PMC11906084; doi:10.1371/journal.pone.0319701)
Supplement: Attachment — Submitted filename: Revision 2 Response to reviewers 2025.docx [file pone.0319701.s017.docx]

**We appreciate the second round of suggestions and hope our answers will clarify the new comments. The answers and changes are highlighted with track changes in the manuscript, and in green in this document.**

**Journal Requirements:

Please review your reference list to ensure that it is complete and correct. If you have cited papers that have been retracted, please include the rationale for doing so in the manuscript text, or remove these references and replace them with relevant current references. Any changes to the reference list should be mentioned in the rebuttal letter that accompanies your revised manuscript. If you need to cite a retracted article, indicate the article’s retracted status in the References list and also include a citation and full reference for the retraction notice.

Thank you for highlighting this issue. After revision, we did find some mistakes in the references. We have now corrected the references and added the DOI to each of them; changes are highlighted along text and also in the references list Lines 471-648.


Reviewers' comments:

Reviewer's Responses to Questions**

**Comments to the Author

1. If the authors have adequately addressed your comments raised in a previous round of review and you feel that this manuscript is now acceptable for publication, you may indicate that here to bypass the “Comments to the Author” section, enter your conflict of interest statement in the “Confidential to Editor” section, and submit your "Accept" recommendation.**

**Reviewer #1: All comments have been addressed**

**Reviewer #2: All comments have been addressed**

**2. Is the manuscript technically sound, and do the data support the conclusions?

The manuscript must describe a technically sound piece of scientific research with data that supports the conclusions. Experiments must have been conducted rigorously, with appropriate controls, replication, and sample sizes. The conclusions must be drawn appropriately based on the data presented.**

**Reviewer #1: Yes**

**Reviewer #2: Yes**

**3. Has the statistical analysis been performed appropriately and rigorously?**

**Reviewer #1: No**

**Reviewer #2: N/A**

**4. Have the authors made all data underlying the findings in their manuscript fully available?

The** [**PLOS Data policy**](http://track.editorialmanager.com/CL0/http:%2F%2Fwww.plosone.org%2Fstatic%2Fpolicies.action%23sharing/1/010f0193d6c1436f-da6e1b0f-38fd-47ae-8b46-069f56edc099-000000/_ASiqMHoeRVSkdSxIk-M9qBC4It4sKbV7NHR41B6T9Y=189) **requires authors to make all data underlying the findings described in their manuscript fully available without restriction, with rare exception (please refer to the Data Availability Statement in the manuscript PDF file). The data should be provided as part of the manuscript or its supporting information, or deposited to a public repository. For example, in addition to summary statistics, the data points behind means, medians and variance measures should be available. If there are restrictions on publicly sharing data—e.g. participant privacy or use of data from a third party—those must be specified.**

**Reviewer #1: Yes**

**Reviewer #2: Yes**

**We included the legend: All analyzed data generated in this study are included in this article and its supplementary information file, in lines 721-722.**

**5. Is the manuscript presented in an intelligible fashion and written in standard English?

PLOS ONE does not copyedit accepted manuscripts, so the language in submitted articles must be clear, correct, and unambiguous. Any typographical or grammatical errors should be corrected at revision, so please note any specific errors here.**

**Reviewer #1: Yes**

**Reviewer #2: Yes**

**6. Review Comments to the Author

Please use the space provided to explain your answers to the questions above. You may also include additional comments for the author, including concerns about dual publication, research ethics, or publication ethics. (Please upload your review as an attachment if it exceeds 20,000 characters)**

**Reviewer #1: The aim of the manuscript is to identify key cellular components of murine duodenal tissue in organoid preparations using digital PCR (dPCR) technique. The results include dPCR results as well as electron microscopic and confocal characterization of duodenal organoids collected at different stages and accordingly classified into early, intermediate and late organoids.
The authors adressed the points raised in the previous round of revision and accordingly changed figures and text in an appropriate manner. The authors discuss that the lack of appropriate statistical analysis and technical replicates derives from the limited biological material. This may be true but still does not justify the description of comparisons between organoid stages as well as the use of terms such as "absolute quantification" in the text. The change of title suggested by another reviewer is in line with this consideration. We think that the use of dPCR as a qualitative exploratory research tool should be more clearly stated throughout the text.**

**Answer: The reviewer correctly mentions that we did not perform an exhaustive statistical analysis because of the lack of technical replications and the limited biological material. To address his concerns, we eliminated the phrase “absolute quantification” to avoid misunderstandings. In addition, we made some changes that can be seen the revised manuscript with track changes in lines 66-71, 224-229, 238-242, 280-281, 408-409,433-434.**

**Also, according to the authors, Fig. 3 and Fig. 4 should be a confirmation of the results obtained by dPCR. This was quite clear for Fig. 4 but it is still less obvious for Fig. 3. Comparisons between different cell populations and specific cellular structures should be more carefully described to justify this figure. At the time being, the figure seems to be inserted to prove the quality of the organoids produced, which is not the aim of the work presented in the manuscript.**

**Answer: Transmission electron microscopy (TEM) plays a crucial role in understanding organoid development because it provides highly detailed, high-resolution images of cellular structures. In our experience, TEM allows us to observe the structural integrity of the different cell populations within organoids as they develop, at better resolution than conventional light microscopy. Additionally, transmission electron microscopy makes it possible to track insights into the tissue architecture, and the structural relationship between different cell types. With the digital PCR characterization of epithelial cell populations in murine duodenal organoids that we are presenting in this article, we must provide evidence to the reader that the cells analyzed are not only functionally but also structurally healthy, through the expression of biomarkers measured by dPCR and confirmed by confocal microscopy for the former, and through TEM for the latter.**

**Reviewer #2: (No Response)**

**7. PLOS authors have the option to publish the peer review history of their article (**[**what does this mean?**](https://track.editorialmanager.com/CL0/https:%2F%2Fjournals.plos.org%2Fplosone%2Fs%2Feditorial-and-peer-review-process%23loc-peer-review-history/1/010f0193d6c1436f-da6e1b0f-38fd-47ae-8b46-069f56edc099-000000/FIY3gX4rs-fJVxMtmLFldaWi-5uleZFnO8aAxanfcfw=189)**). If published, this will include your full peer review and any attached files.


If you choose “no”, your identity will remain anonymous but your review may still be made public.


Do you want your identity to be public for this peer review? For information about this choice, including consent withdrawal, please see our** [**Privacy Policy**](https://track.editorialmanager.com/CL0/https:%2F%2Fwww.plos.org%2Fprivacy-policy/1/010f0193d6c1436f-da6e1b0f-38fd-47ae-8b46-069f56edc099-000000/s0vq5LWUIVnFuA4hfBjSvFO0ltULKcYdRdwSkMy-qpA=189)**.**

**Reviewer #1: No**

**Reviewer #2: Yes: Huda Hisham Sultan Alkatib**
